# A stochastic contact network model for assessing outbreak risk of COVID-19 in workplaces

**DOI:** 10.1371/journal.pone.0262316

**Published:** 2022-01-14

**Authors:** Xi Guo, Abhineet Gupta, Anand Sampat, Chengwei Zhai

**Affiliations:** One Concern, Inc., Menlo Park, CA, United States of America; VIT University, INDIA

## Abstract

The COVID-19 pandemic has drastically shifted the way people work. While many businesses can operate remotely, a large number of jobs can only be performed on-site. Moreover as businesses create plans for bringing workers back on-site, they are in need of tools to assess the risk of COVID-19 for their employees in the workplaces. This study aims to fill the gap in risk modeling of COVID-19 outbreaks in facilities like offices and warehouses. We propose a simulation-based stochastic contact network model to assess the cumulative incidence in workplaces. First-generation cases are introduced as a Bernoulli random variable using the local daily new case rate as the success rate. Contact networks are established through randomly sampled daily contacts for each of the first-generation cases and successful transmissions are established based on a randomized secondary attack rate (SAR). Modification factors are provided for SAR based on changes in airflow, speaking volume, and speaking activity within a facility. Control measures such as mask wearing are incorporated through modifications in SAR. We validated the model by comparing the distribution of cumulative incidence in model simulations against real-world outbreaks in workplaces and nursing homes. The comparisons support the model’s validity for estimating cumulative incidences for short forecasting periods of up to 15 days. We believe that the current study presents an effective tool for providing short-term forecasts of COVID-19 cases for workplaces and for quantifying the effectiveness of various control measures. The open source model code is made available at github.com/abhineetgupta/covid-workplace-risk.

## Introduction

The current pandemic of COVID-19 has now led to more than 180 million cases and more than 3.9 million deaths worldwide as of 2021–06-30 [1]. The disease is caused by SARS-CoV-2—a type of respiratory virus. Compared to other coronaviruses like severe acute respiratory syndrome coronavirus (SARS-CoV) and the Middle East respiratory syndrome coronavirus (MERS-CoV), it is less deadly but has higher transmissibility [2]. Due to its airborne nature, long incubation, and heightened transmissibility, the pandemic has endangered public health, especially those of workers in various types of settings. For example, there has been a considerable number of outbreaks in meat processing facilities [3–5], offices, warehouses, and university campuses [6–9].

Amidst this pandemic, government and businesses have taken significant measures to mitigate the outbreak risk in indoor workplaces including capacity reduction, remote working, mask wearing policy, social distancing, etc. The percentage of employers who reported a successful shift to remote work have increased from 73% to 83%, from June 2020 to January 2021 [10]. However, more than 60% of workers in the United States economy cannot work remotely and this percentage is even higher in emerging economies [11]. For those able to work remotely, a survey of executives suggests that COVID-19 may propel faster adoption of automation and artificial intelligence [12], work from home may increase from 20% pre-pandemic to 27% post-pandemic [13], and hybrid work arrangements with employees splitting time at home and in the office will be the new normal [10, 14].

While the workforce is adapting to these significant changes, the need for ensuring the safety of workers in the long-term is becoming more urgent. Business owners are concerned about reoccupying their workplaces, but their expectations are far from reality. In June 2020, 80% of executives expected a return back to normal by September 2020, and 88% by December 2020 but most of these businesses continue being fully remote even in April 2021 [15]. Meanwhile, executives have been hard-pressed to make the right mitigation decisions for safe return of their employees. In a survey of 100 executives from June 2020, 78% had implemented or planned to implement once a day temperature checks while only 26% favored contact tracing and 11% favored regular testing of employees, showing a preference for more convenient measures like temperature checking which is less effective due to the prevalence of pre-symptomatic and asymptomatic cases [15]. At the same time, information about COVID-19 transmission has continued to evolve making it difficult for businesses to choose the right mitigation measures to maximize safety and minimize disruptions. Businesses need to make near real-time decisions in response to local trends of disease circulation while being resilient against long-term changes in the pandemic. In order to make effective decisions for reoccupation of workplaces, facility managers, human resources personnel, and business decision makers need data-driven tools to help them assess outbreak risks within their facilities based on local community conditions, and the relative benefits of different mitigation measures.

There are several differences between modeling disease dynamics in a large population and modeling transmissions in a workplace with a small number of people. Parameters such as probability of transmission (*β*, *R*_0_) are relatively easy to estimate in a large population compared to smaller groups of employees in workplaces. In indoor spaces with restricted access such as offices and warehouses, population is generally static, and properties of the environment and individual behavior have large impacts on the transmission [16, 17]. Furthermore, an infectious pathogen can often invade and be sustained in a large population when *R*_0_ > 1, but in small and closed populations, the disease transmission can die off quickly due to either the depletion of susceptible population or removal of infectious population [18]. Due to these differences, the traditional compartmental models often do not fit the purpose of modeling the risk of outbreaks in small and closed population.

Several recent models have aimed to simulate the physics behind COVID-19 transmission within offices, classrooms, and restaurants [19, 20]. These studies estimate the transmission risk at the point of the transmission event, but do not provide estimation on the scale of outbreaks. Other studies have modeled human behavior in indoor spaces using agent-based or network-based models [21–23]. For example, a contact network model was developed to estimate the cumulative incidence of COVID-19 on the Princess Diamond Cruise ship [21]. These models utilize the unique contact network structure in their specific cases, like the interactions between passengers, interaction between crew members, and interactions between passengers and crew members on a cruise ship. However, they are difficult to generalize for other locations and settings.

Recently, several models and web-based tools have also been developed to model outbreaks in large gatherings or indoor places [24–26]. The majority of these models are deterministic. A web-based tool called the COVID-19 Indoor Safety Guideline aims to develop safety guidelines based on room specifications, human behavior, age group, and virus variant [25]. The study demonstrated the impact of airflow, human behavior, and indoor environment on SARS-CoV-2 transmission, but it did not consider the impact of local incidence rate on the introduction of the outbreaks. Another application is developed to estimate the risk that at least one individual with SARS-CoV-2 is present in gatherings of different sizes in the United States [27]. Their assumption of community introduction following Bernoulli trials matches that of the present study. However, the tool does not provide the ability to assess outbreak risk in workplaces. An agent based stochastic model called Covasim has been created to project epidemic trends, explore intervention scenarios, and estimate resource needs [22]. The model is composed of a collection of open-source data-driven synthetic contact networks, like SynthPops and hybrid networks. However, the study did not include information about its applicability for assessing risks in workplaces with small population.

This work contributes to the body of knowledge in modeling COVID-19 outbreaks in indoor places. It incorporates secondary attack rate (SAR) as the transmission parameter into a stochastic contact network. By consistently accounting for uncertainties in the transmission chain, this model enables probabilistic short-term forecasting of the outbreak risk within workplaces with static populations. It also allows for customization based on local case rates, control measures such as mask wearing, behaviors of employees such as interactions and speaking activity, and environmental factors such as airflow and filtration. Additionally, we provide guidance for parameter values appropriate for general workplaces so that the model can be implemented widely.

The objectives of this study include—presenting a contact-network model that can be used to forecast cumulative incidences of COVID-19 in small populations; selecting probabilistic parameters associated with transmission dynamics and control measures to evaluate the impact of mitigation; and validating the model with real-world examples. The design and methodology of the stochastic contact-network model is presented in the Materials and Methods section. The validation against observations from 7 individual outbreak reports in workplaces worldwide, and against weekly reported data from over 8000 nursing homes in the United States is presented in the Results section. The observations closely match model estimates for both sets of comparisons. In the Discussion section, we highlight the primary limitations of the work, and motivations for future work. Finally, we present a summary of our conclusions.

## Materials and methods

We present a stochastic contact network which emulates the introduction of COVID-19 cases in a facility and the daily contacts among people within the facilities as the routes of transmission. The nodes in the network represent each individual, and the edges represent their interactions or contacts. The occurrence of edges is stochastic and homogeneous. We only consider the infectious, susceptible, and exposed populations in the model. The model outputs the cumulative incidence within a facility after *n* days, where *n* ≤ 15 is intended to be a short forecasting period.

### Network structure

Our model adopted a star network topology [28, 29] to represent the contacts between the first generation and the susceptible employees. The central hub of the network represents the first-generation cases who arrive at the workplace. Depending on the local case rates, there could be one or several hubs, hence one or several independent star networks. Moreover, new case introductions can occur during the forecast period, hence new hubs may be added on each day. The edges connecting the central hub and other nodes indicate contact made between the first-generation cases and the susceptible employees on site. Each hub draw connections with nodes randomly based on a geometric distribution. The edges can be created for each day, remain for the next day, or only exist for one day depending on the probability of contacts being maintained for the next day. Successful transmission of the pathogen through edges (contacts) is governed probabilistically by the secondary attack rate. Networks for each first-generation case are mutually independent.

### Model design

To model the transmission dynamic within a short-term period, we defined the components of a transmission chain as below.

- **Case introduction**—Introduction of COVID-19 cases in a facility is defined as the time when the first case arrived in the facility.
- **Transmission chain**—The process in which the virus spreads from one person to another. In our contact network, the transmission chain is represented by series of nodes (infectious, susceptible, and exposed individuals) and edges (contacts).
- **First-generation cases**—Individuals who are the starting point of a transmission chain. These include one or more index cases that are infected outside a facility and introduce the disease into the facility. This group is considered as the infected population.
- **Second-generation cases**—Individuals who are exposed to COVID-19 as a result of a successful transmission event after coming into contact with the first-generation cases. This group forms the exposed population. In the present model, we assume that these individuals do not become infectious within the short forecasting period. Hence, the transmission chain ends at second-generation cases.

The pseudocode in Algorithm 1 illustrates the design of the model. The first-generation cases who are infected from the community can arrive at the facility on any given day. We assume that these cases are introduced to the facility as Bernoulli trials using local daily new case rate as the success rate. Once first-generation cases arrive, we initiate random contacts between the infectious and the susceptible individuals. The daily contact size of the first-generation cases is randomly determined from a geometric distribution based on the average daily contacts. The employees contacted by the first-generation case(s) are randomly chosen from the population. Additionally, the model has a parameter to probabilistically determine how many contacts remain as contacts for the next day.

The transition from susceptible to exposed state takes place among the employees who make contacts with the first-generation cases based on the secondary attack rate (SAR). A randomized SAR drawn from a Beta distribution is assigned to each first-generation case. The average SAR for a workplace is determined based on mask compliance, airflow, filtration, speaking volume, and speaking percentage.

The required number of simulations to achieve convergence for the model depends on the parameter values. For a wide range of parameters, we found that the average cumulative incidence converges at *n*_sim_ ≥ 1000.

**Algorithm 1 Model Pseudocode**. Stochastic contact network model for short-term forecasting within facilities

**Require**:

 *p*_case_← daily new case rate

 *e*← total employees or nodes

 *n*_days_← total number of modeling days

 *α*_SAR_← alpha parameter for SAR Beta distribution

 *β*_SAR_← beta parameter for SAR Beta distribution

 $\bar{c}$ ← average daily contact size

 *p*_remain_← probability that edges (contacts) are sustained for the next day

 *n*_sim_← number of simulations

 **for all** sim ∈ *n*_sim_ **do**

  Sample number of first-generation cases for *n*_days_, $n_{g1}≔(g1^{\left(1\right)},\ldots ,g1^{\left(n_{\text{days}}\right)})\sim \text{Binomial}(e,p_{\text{case}})$

  Initialize empty array of node indices for first-generation cases, ***g*1** ≔ ()

  **for all** *g*1 ∈ ∑ ***n*_*g*1_** **do**

   Sample SAR for each first-generation case, SAR_*g*1_ ∼ Beta(*α*_SAR_, *β*_SAR_)

   Initialize empty array of previous day’s contacts for each first-generation case prior to day 1, $c\_remain_{g1}^{\left(0\right)}≔\left(\right)$

  **end for**

  **for all** *d* ∈ *n*_days_ **do**

   Sample unique indices of first-generation cases for day *d*, ***g*1** = ***g*1** + *Uniform*(*e*, *g*1^(*d*)^)

   **for all** *g*1 ∈ ***g*1 do**

    Sample number of contacts for each first-generation case for day while ensuring they do not exceed total employees $n_{c_{g1}}^{\left(d\right)}∼\text{min}(\text{Geometric}(\frac{1}{\bar{c}+1}),e)$

    Sample indices of new contacts, $c\_new_{g1}^{\left(d\right)}∼\text{Uniform}\left(e,n_{c_{g1}}^{\left(d\right)}-\text{size}\left(c\_remain_{g1}^{\left(d-1\right)}\right)\right)$

    Total secondary contacts for day, $c_{g1}^{\left(d\right)}≔c\_new_{g1}^{\left(d\right)}+c\_remain_{g1}^{\left(d-1\right)}$

    Sample indices of second-generation cases from contacts for day *d*, $g2^{\left(d\right)}∼\text{Binomial}\left(c_{g1}^{\left(d\right)},{\text{SAR}}_{g1}\right)$

    Sample contacts that remain consistent the next day, $c\_remain_{g1}^{\left(d\right)}∼\text{Binomial}\left(c_{g1}^{\left(d\right)},p_{\text{remain}}\right)$

   **end for**

  **end for**

  Total cumulative incidence, cases_sim_ ≔ Unique(***g*1** + ∑_*d*_ ***g*2^(*d*)^**)

**end for**

### Model parameters and implementation

This section describes the model implementation, and the distribution of model parameters.

#### Case introduction

We assume that first-generation cases are introduced into a facility as Bernoulli trials using local daily new case rate as the success rate. Therefore, the probability of *x* infectious employees arriving on any given day at a facility with *e* employees, and the local daily new case rate *p*_case_, *P*(*x*) is calculated from a Binomial distribution as *P*(*x*) = Binomial(*x*, *e*, *p*_case_). Because of the short modeling period, and possibility of test lagging and underreported cases, we assumed the case rate to be constant throughout the modeling period, although a variable case rate can also be implemented. The assumption of Binomial distribution matches similar approaches used in other studies for modeling probability of case occurrence [24, 27, 30–32].

#### Secondary attack rate (SAR)

Secondary attack rate (SAR) is defined as the probability of an infection being transferred to a naive individual [33]. It is approximated as the proportion of secondary cases induced in a contact group by the first case in the group [34–36]. In the present model, it is the probability of getting exposed among all secondary contacts of a first-generation case.

Multiple studies on COVID-19 outbreaks have reported a wide range of SAR across different settings [37–43]. High values of SAR have been observed in specific settings, such as—SAR = 53.3% during loud singing [39], SAR = 84.6% after a business meeting [44], and SAR = 38.8% when having meals together [45]. A meta-analysis of SAR for COVID-19 concluded that workplaces where close contacts are less intense and frequent tend to have lower values of SAR (0%–5.3%) than household settings (3.9%–54.9%) [43]. These values of SAR reflect transmission in naive populations, and we have not considered the reduction in transmission due to COVID-19 vaccination in this study.

The SAR associated with each infectious individual varies because of their viral load and course of infection [43, 46]. We incorporate this variability by sampling SAR for each first-generation case from the Beta distribution. The SAR assigned to each first-generation case remains constant throughout the forecast period as a simplifying assumption. The Beta distribution is selected because it is defined on the desired interval [0, 1] and is able to model long tails. The distribution has two parameters: *α*_SAR_ and *β*_SAR_. We expect the SAR distribution in a population to be long-tailed, where a majority of infectious individuals exhibit low SAR or infect few people, while a minority of individuals exhibit high SAR [43, 47]. A long-tail distribution can be implemented in the Beta distribution by setting *α*_SAR_ = 1. Then, the *β*_SAR_ is calculated from the average $\bar{\text{SAR}}$ across multiple individuals, as shown below.

$$\begin{array}{c} \beta_{\text{SAR}}=\alpha_{\text{SAR}}\frac{1-\bar{\text{SAR}}}{\bar{\text{SAR}}} \end{array}$$ (1)

Multiple studies have concluded that the secondary attack rates associated with the transmission of airborne pathogens vary based on particle emission and inhalation rates, airflow, and filtration [19, 25, 48]. Here we present an approach to modify $\bar{\text{SAR}}$ in a facility based on these parameters.

Based on one of the studies [19], and the assumptions described in S3 Appendix, the average SAR for airborne transmission of COVID-19 depends on inhaled virions as -

$$\begin{array}{cc} \bar{\text{SAR}} & =1-\text{exp}(-N^{\prime }) \end{array}$$

and

$$\begin{array}{cc} N^{\prime } & \propto \frac{S(1-m_{e})}{\lambda }\\ \Rightarrow \frac{N_{i}^{\prime }}{N_{j}^{\prime }} & =\frac{S_{i}\left(1-m_{e_{i}}\right)}{\lambda_{i}}\frac{\lambda_{j}}{S_{j}\left(1-m_{e_{j}}\right)} \end{array}$$ (2)

where subscripts *i* and *j* represent any two sets of parameters. *N*′ is the total number of inhaled virions normalized by the average infectivity threshold. *m*_*e*_ ∈ [0, 1] is the mask effectiveness. *S* is the virion emission at the source and is a function of speaking percentage and volume. λ is the decay rate of virions and is a function of airflow and filtration.

*Source emission rate*. We assume that the source emission rate *S* is a function of speaking percentage *s*_*p*_, and speaking volume *s*_*v*_ in decibels. It was observed from case reports that talking releases 46 times more virions than breathing [19]. Other studies have concluded that higher volume releases higher number of aerosolized particles carrying virions [25, 48]. We represent the increase in virions based on volume by the volume multiplier *s*_*m*_. Then, source emission *S* is described as -

$$\begin{array}{c} S=\left(1-s_{p}\right)+46s_{p}s_{m} \end{array}$$ (3)

Based on measurements from 10 participants, increasing the speaking volume from 70 dB to 98 dB increased the particle emission rate from 6 to 53 particles per second [48]. Additionally, the particle emission rate is directly proportional to the root mean square amplitude of the vocalization. Since the decibel level depends on the logarithm of amplitude, the relationship with emitted particles or virions *q* can be described as -

$$\begin{array}{cc} s_{v} & \propto \text{log}(q)\\ \Rightarrow s_{v} & =C_{q}\text{log}\left(q\right) \end{array}$$ (4)

where *C*_*q*_ is an unknown constant, and using the above values, is computed as -

$$\begin{array}{c} C_{q}=\frac{98-70}{\text{log}(53/6)} \end{array}$$ (5)

Assuming that the ratio of 46 virions corresponds to speaking at a reference volume of 60 dB, the activity multiplier *s*_*m*_ at an arbitrary volume level can then be computed as -

$$\begin{array}{c} s_{m}={\frac{53}{6}}^{\left(s_{v}-60\right)/\left(98-70\right)} \end{array}$$ (6)

*Decay rate*. The decay rate λ is a function of air exchange rate or airflow λ_*a*_ (air changes per hour or ACH), viral settling and deactivation λ_*d*_ = 0.62/h, and decay due to filtration λ_*f*_ [19].

$$\begin{array}{c} \lambda =\lambda_{a}+\lambda_{d}+\lambda_{f} \end{array}$$ (7)

*Mask effectiveness*. The mask effectiveness parameter *m*_*e*_ combines the contribution of masks from both source emission rate and breathing rate, and effectively reduces the number of inhaled virions as shown in Eq 2. Based on a meta-analysis on the impact of mask-wearing on COVID-19 transmission, the relative risk (RR) of mask wearing under non-healthcare setting is RR = 56%, and for healthcare setting is RR = 30% [49]. Then mask effectiveness is implemented in Eq 2 as the factor *m*_*e*_ = 1 − RR. More research is needed to better characterize mask effectiveness, and studies have shown high variability of mask effectiveness for cloth masks [50].

*Reference SAR*. In order to use Eq 2 to estimate $\bar{\text{SAR}}$ for any set of speaking percentage, volume, airflow, filtration, and mask effectiveness, we need a reference ${\bar{\text{SAR}}}_{\text{ref}}$ and its corresponding parameters as shown in Eq 8.

$$\begin{array}{cc} \bar{\text{SAR}} & =1-\text{exp}(-N^{\prime })\\ N_{\text{ref}}^{\prime } & =-\text{log}(1-{\bar{\text{SAR}}}_{\text{ref}})\\ \frac{N^{\prime }}{N_{\text{ref}}^{\prime }} & =\frac{S(1-m_{e})}{\lambda }\frac{\lambda_{\text{ref}}}{S_{\text{ref}}\left(1-m_{e_{\text{ref}}}\right)} \end{array}$$ (8)

**Fig 1:**
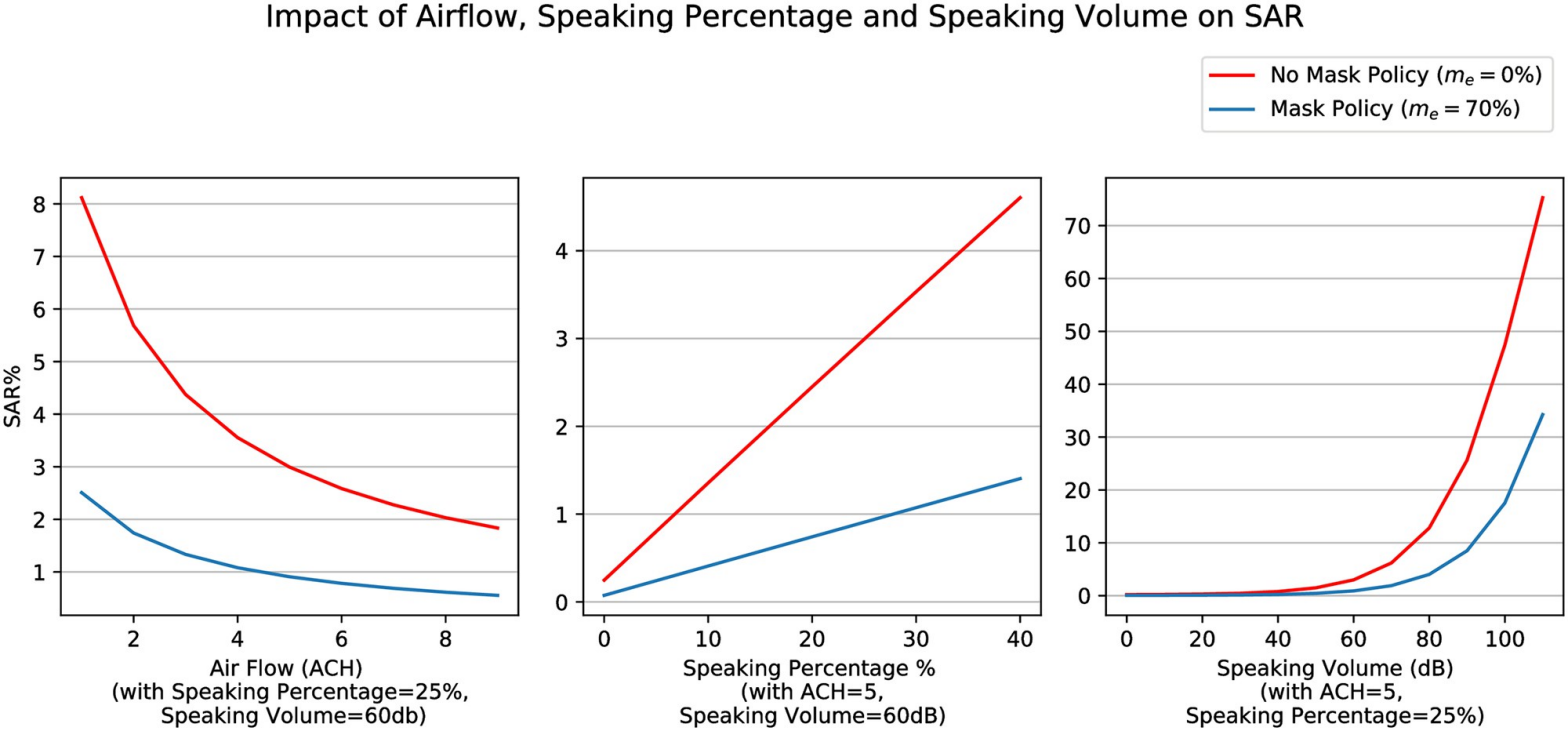
$\bar{\text{SAR}}$ variation. Impact of airflow, speaking volume, speaking percentage, and mask effectiveness on SAR.

Different studies have presented SAR among non-household contacts in the range of 0%-5.3% [37, 42, 43, 51, 52]. In this study, we assume a reference SAR, ${\bar{\text{SAR}}}_{\text{ref}}=5.1\%$ as the one associated with non-household unprotected contacts from the Germany outbreak study in a workplace setting [37], and assume the following associated parameters—airflow λ_*a*_ = 2 ACH, filtration λ_*f*_ = 0, speaking percentage *s*_*p*_ = 25%, speaking volume *s*_*v*_ = 65 dB, and mask effectiveness *m*_*e*_ = 0. Fig 1 shows the resulting variation of SAR for different parameters. Determination of these parameters is based on expert judgement as little information exists in literature. The assumptions for selection of these parameters are described in S1 Appendix.

#### Daily contact size

Daily contact size is defined as the number of secondary contacts of each first-generation case on each day, and are randomly sampled from a Geometric distribution supported on *x* ≥ 0. Given the average daily contacts $\bar{c}$, the probability of daily contacts *P*(*c* = *x*) is defined as -

$$\begin{array}{cc} P(c=x) & =\text{Geometric}(x,\frac{1}{\bar{c}+1})\;\forall \;x\geq 0\\ & ={\left(1-\frac{1}{\bar{c}+1}\right)}^{x}(\frac{1}{\bar{c}+1}) \end{array}$$ (9)

The parameterization of the Geometric distribution is based on data collected by the citizen science-based BBC pandemic study [53, 54]. The workplace contacts in the BBC study align well with data from an independent POLYMOD study [55, 56]. Based on the BBC study, the average daily contacts at work are $\bar{c}=6$. The data shows that daily workplace contacts are long-tailed and hence can be modeled with the Geometric distribution.

Additionally, we expect that employees contact some of the same people every day at work. The model incorporated this using a parameter for the probability that contacts remain the same the next day *p*_remain_. We assumed *p*_remain_ = 60% based on an office study in Italy [57].

#### Preventive and control measures

The contact network model presented above can be effectively used to assess return-to-work policies in workplaces. Some examples of control measures and their impacts on the model are presented below and in Fig 1.

- **Work from home**—When a percentage of employees work remotely, it reduces the number of employees *e* in the workplace. This reduces the number of case introductions, and hence cumulative incidence. With some employees working remotely, contacts could also be reduced by the same proportion.
- **Mask wearing**—Masks reduce both the source emission and the breathing rate for virions, and is implemented in the model using the parameter *m*_*e*_. Wearing masks reduces the number of virions *N*′, thus reducing average SAR, and hence cumulative incidence.
- **Ventilation improvement**—Increasing airflow and incorporating filtration increases the decay rate λ, resulting in reduction in average SAR.
- **Vocal loudness and speaking percentage**—Decreasing vocal loudness and the amount of talking leads to lower amounts of emitted virions *S*. This in turn reduces average SAR.
- **Social distancing**—Social distancing reduces the average daily contacts $\bar{c}$ in the model, thus reducing cumulative incidence.

### Individual outbreak reports from workplaces for model validation

One of the datasets used for validating our stochastic model was epidemioloigcal reports on COVID-19 outbreaks in workplace-like situations, as shown in Table 1. Generally, information on environmental settings of these outbreaks were not reported, hence instead of selecting a single parameter value, we selected a range based on expert judgement. Table 1 summarizes the collected outbreaks and the parameters associated with them in the model. For the Henan Expressway outbreak, because the employees share the same residence, we used a fixed SAR = 20% which is similar to a household SAR, hence some of the inapplicable parameters are listed as N.A. in the table. Further description of each outbreak and the parameter selection process is provided in S1 Appendix.

**Table 1:** Outbreak reports for workplace outbreaks. Model parameters associated with outbreak reports used for validation.

Outbreak Name	*e*	*p*_case_ (per 100k)	$\bar{c}$	λ_*a*_ (ACH)	*s*_*v*_ (dB)	*s*_*p*_ (%)	*n*_days_	*p*_remain_
1. Tianjin Office [58]	906	1	4–10	1.5–4	65	5–25	10	0.4
2. Korean Call Center [8]	216	1	4–10	1.5–4	70	55–66	14	0.4
3. San Diego VA Office [9]	100	1	4–10	1.5–4	65	5–40	8	0.4
4. Singapore Conference [59]	111	1	6–12	1.5–4	70	25–40	3	0
5a. South Dakota Meat Processing Plant (First Shift) [5]	1744	1	4–10	1–11	70	5–20	14	0.4
5b. South Dakota Meat Processing Plant (Second Shift) [5]	1459	1	4–10	1–11	70	5–20	14	0.4
6. Henan Expressway [60]	103	1	2–4	N.A.	N.A.	N.A.	13	0.4
7a. Major League Baseball Team with Social Distancing and Mask Protocols [61]	68	60	4–8	1.5–4	65	5–25	10	0.4
7b. Major League Baseball Team without Social Distancing and Mask Protocols [61]	68	60	4–10	1.5–4	65	5–25	10	0.4

For validation, the cumulative incidence from the model were compared with the observations. For each outbreak, simulation starts from when the case introduction was reported. Hence, we simulated cumulative incidence only for that subset of simulations with at least one case on the first day of the modeling period. We generated two model estimates for each outbreak—lower bound and upper bound. To generate lower bound estimates, we selected the lower bound of average daily contacts and speaking percentage, and the upper bound of airflow, since this combination results in the lowest cumulative incidence; and vice-versa for the upper bound estimates. For instance, for the Tianjin Office outbreak, the lower bound model simulation is generated by the minimum average daily contact size $\bar{c}=4$, the minimum speaking percentage *s*_*p*_ = 5%, and the highest airflow λ_*a*_ = 4 ACH. Finally, if incidences were recorded on a daily basis in the reports, we additionally compared the temporal trends.

### Weekly cases from nursing homes for model validation

Another dataset used for validating our model is the weekly COVID-19 data in nursing homes compiled and published since May 2020 by the Division of Nursing Homes; the Quality, Safety, and Oversight Group; and the Center for Clinical Standards and Quality at the Centers for Medicare and Medicaid Services [62].

For our analysis, we considered data associated with facilities prior to, and including the week when the first confirmed cases were reported. There are 8423 unique facilities in 2463 unique counties in the dataset for the analyzed period, totalling 89273 data entries. We assumed that resident population in a facility is equivalent to the number of beds, and staff population is 90% of the number of residents [63]. We also assumed that only staff population may be first-generation cases in nursing homes since in general, the resident population is expected to remain within the facility, while the staff population attends the facility each day similar to a workplace. We incorporated this as a modification to our stochastic model such that first-generation cases are sampled only from the staff population. We assumed the number of daily interactions in nursing homes is $\bar{c}=6$ average contacts per person per day, and the average SAR is $\bar{\text{SAR}}=5.1\%$. Relevant data attributes from the dataset, and data processing steps are further described in S2 Appendix.

## Results

In this section, we validate our model by comparing model estimates against observed data from outbreaks of COVID-19 in workplaces, and nursing home facilities.

### Comparisons with outbreak reports in workplaces

Table 2 summarizes the comparisons between the observations and the simulated cumulative incidences. The model outputs include the minimum and maximum cumulative incidence (shown as Range) across all simulations, the interquartile range (25th to 75th percentile), the median, and the percentile of the observation within the predicted range from both the lower bound and the upper bound models. All observations are within the ranges of model simulations, except for the Korean Call Center, the first shift of the South Dakota Meat Processing Plant, and the Major League Baseball Team outbreaks where observations exceed the lower bound estimates. Figures showing comparisons of temporal trends for 3 of the outbreaks are included in S1 Appendix. Overall, the current model captures the case growth rate within 14 days post case introduction for all outbreaks. The daily observations lie within the range of the model simulations except for a few days in the Korean Call Center outbreak where the lower bound model simulations are higher than the observations. The comparisons demonstrate that all observations are within the range of cumulative incidences estimated from the upper bound parameters in the current model. This is expected since the real-world outbreaks collected here are mostly large-scale non-familial cluster cases, while sporadic cases are usually not reported. As a result, we believe that the contact network model presented in this study is reasonable for modeling short-term cumulative incidences in workplaces.

**Table 2:** Observations and model estimates for workplace outbreak reports.

Outbreak Name	*I*_obs_	Lower Bound Model				Upper Bound Model			
		%-ile	range	IQT	median	%-ile	range	IQT	median
1. Tianjin Office	7	99.9	1–9	1–2	1	58.9	1- 55	3–10	5
2. Korean Call Center	76	N.A.	1–34	2–8	4	97.9	1–135	10–38	22
3. San Diego VA Office	5	99.9	1–5	1–1	1	45	1–55	3–11	6
4. Singapore Conference	7	99	1–12	1–2	1	74	1–55	2–8	4
5a. SD Meat Processing (First Shift)	32	N.A.	1–7	1–2	1	88.9	1–102	5–21	12
5b. SD Meat Processing (Second Shift)	6	99.9	1–7	1–2	1	26.7	1–108	5–21	11
6. Henan Expressway	6	60	1–30	2–8	5	36	1–51	4–14	8
7a. Major League Baseball Team with Social Distancing and Mask Protocols	20	N.A.	1–8	1–2	1	99.8	1–28	2–6	4
7b. Major League Baseball Team without Social Distancing and Mask Protocols	20	N.A.	1–9	1–2	1	95.2	1–43	3–10	6

Observations and model outputs of cumulative incidence for outbreaks with *n*_sim_ = 5000 for each model. *I*_obs_ refers to the observed cumulative incidences;%-ile refers to the percentiles where the observations fall within the predicted range and N.A. indicates the observation is outside the range; IQT refers to the interquartile, or the 25th to 75th percentile range of the model output.

### Comparisons with weekly cases from nursing homes

In this section, we present the comparison of the model simulations with observations from the nursing home facilities. The comparisons were made for probability of case introduction, average 7-day cumulative incidence, and the distribution of cumulative incidence. Each run of the model included *n*_sim_ ≥ 10000 simulations to ensure convergence of the outputs. Overall, the model estimates agreed well with the observations from nursing homes, and are described in detail below.

#### Probability of case introduction

We first compared the observed probability of case introduction in a facility with our model estimate. Since the nursing homes data is reported on a weekly basis, the estimated probability of at least one case being introduced in the facility in 7 days, *P*_*est*_(*x* ≥ 1∣*t* = 7) is only dependent on the number of people in a facility, and the local case rate, and is computed as -

$$\begin{array}{c} P_{\text{est}}\left(x\geq 1|t=7\right)=1-{\left({\left(1-p_{\text{case}}\right)}^{e_{s}}\right)}^{7}, \end{array}$$ (10)

where *e*_*s*_ is the number of staff in each nursing home, and *p*_case_ is the daily new case rate in the county. As mentioned in the Materials and Methods section, the following analysis only considered data associated with facilities prior to, and including the week when the first confirmed cases were reported.

In order to calculate observed probabilities of case introduction, the data needs to be divided into groups. Since the estimated probability is a function of the local case rate, we binned the data by the county-level 7-day mean case rate and generated probabilities within each bin. The data were categorized into 40 bins with approximately 2200 data entries in each bin. Each entry corresponded to one facility for one week. Note that the same facility over different weeks can be categorized into different bins based on the case rate in the county for each corresponding week. Then within each bin, the probability of at least one case introduction, *P*_*obs*_(*x* ≥ 1∣*t* = 7) is computed as -

$$\begin{array}{c} P_{obs}\left(x\geq 1|t=7\right)=\frac{n_{x_{bin}\geq 1}}{N_{bin}}, \end{array}$$ (11)

where $n_{x_{bin}\geq 1}$ is the number of data entries in a given bin with at least one confirmed case, and *N*_*bin*_ is the total number of entries in the bin.

**Fig 2:**
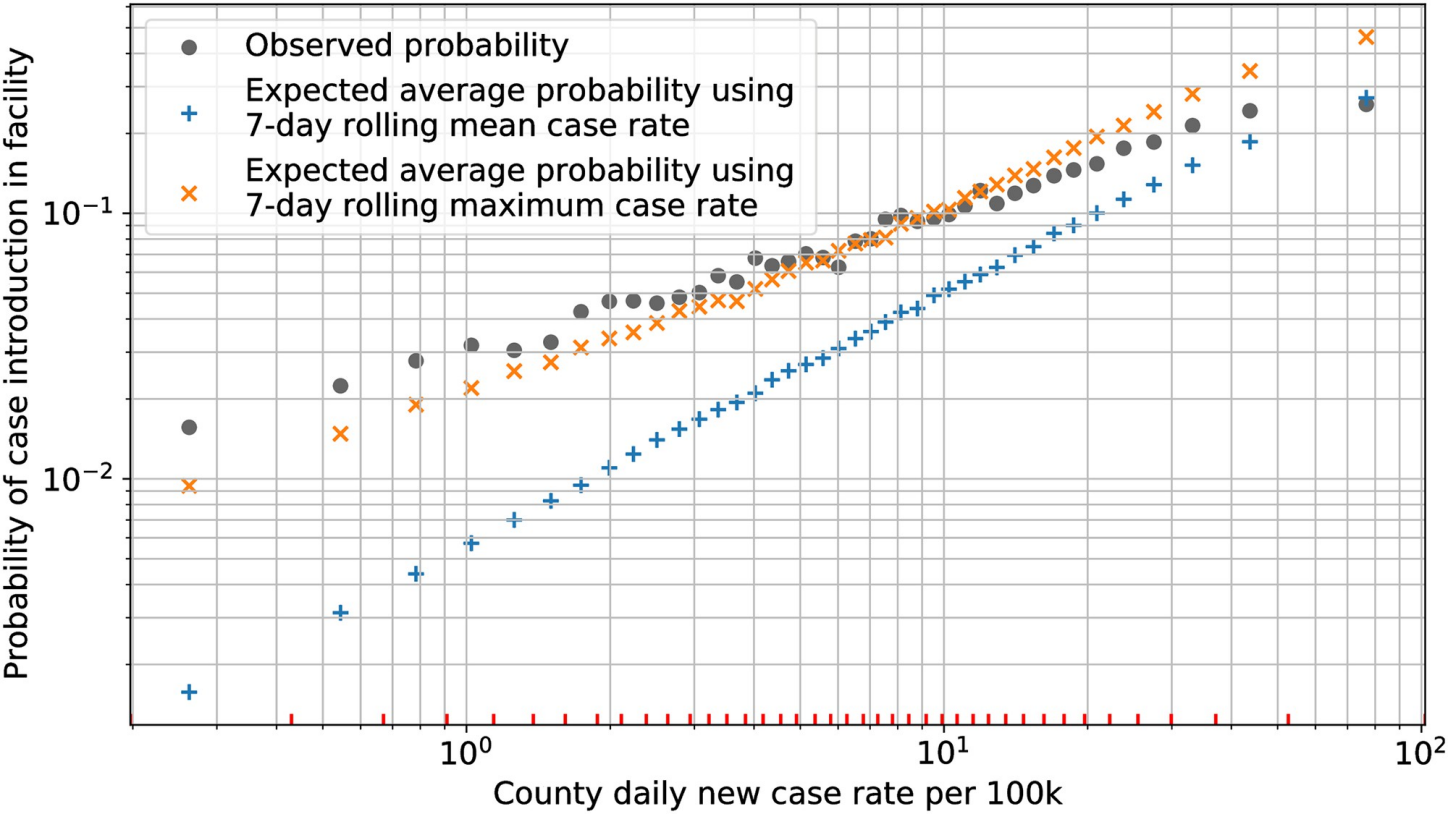
Probability of COVID-19 case introduction. Comparison of observed and expected probability of COVID-19 cases getting introduced in nursing homes based on the county-level case rates. Observations include all weeks prior to and including when the case introduction was reported in each facility. The red ticks on the x-axis mark the 7-day mean case-rate boundaries of each category bin.

Fig 2 shows the comparison of the estimated probability, with the observed probability of case introduction in a facility within 7 days. The abscissa is for representation only and marks the average case rate within each bin. The estimated probabilities were computed separately for each facility and week, and then averaged across all data entries in each bin. Additionally, the estimated probability was computed using two different case rates for each data entry—the 7-day mean case rate, and the 7-day maximum case rate. The former represents the average of the daily new cases per 100,000 population during the previous 7-days from the reporting date of the facility, while the latter similarly represents the maximum.

We observe in Fig 2 that the observed probability of case introduction varies approximately linearly on the log-log scale with the county case rate. This matches the expected behavior for the Binomial distribution as presented in Eq 10, and hence corroborates that the assumption for Binomial distribution for case introduction is reasonable.

We also observe in the figure that the observed probability is higher than estimated probability when using the 7-day mean case rate, and matches closely with the estimate based on 7-day maximum case rate. In the dataset, the average 7-day maximum case rate within a category bin could be up to 6 times higher than its counterpart mean case rate. Since most of the cases were observed in nursing homes in the early stage of the pandemic, this comparison suggests that case rates in the United States could be under-reported due to limited testing capacity [64, 65]. Other factors contributing to the differences between observations and estimates could be our assumptions about the population in nursing homes, and that cases are introduced in facilities only from the staff. Test lagging, long incubation periods, and reporting delays could also contribute to these differences.

#### Average 7-day cumulative incidence

The probability of case introduction is only dependent on local case rates and population size, and does not impact the dynamics of in-facility transmission. In this section, we compare the observed weekly cases in facilities with the model estimates. The observations represent cases from both case introductions and in-facility transmission, and these contributions cannot be differentiated.

The same dataset and binning methodology was used as described for the previous comparison. Within each bin, the average of confirmed cases across all data entries was calculated. This average encompasses the zero cases observed in facilities during the weeks prior to their respective case introduction. This average value represents the average of observed 7-day cumulative incidence in the nursing homes corresponding to each category bin.

For each bin, a model was run with a case rate calculated using the geometric mean of the case rates across all data entries in the bin. The geometric mean is equivalent to taking the average in log-space and was selected because it is more robust when bin sizes are large, mainly in the first and the last category bins. Since each facility in a category bin can have a different population, the average number of beds across all data entries in a bin was used as an approximation for the average number of residents in a facility for that bin. For each bin, the average number of estimated cases was calculated by taking the average across all model simulations of the 7-day cumulative incidence.

**Fig 3:**
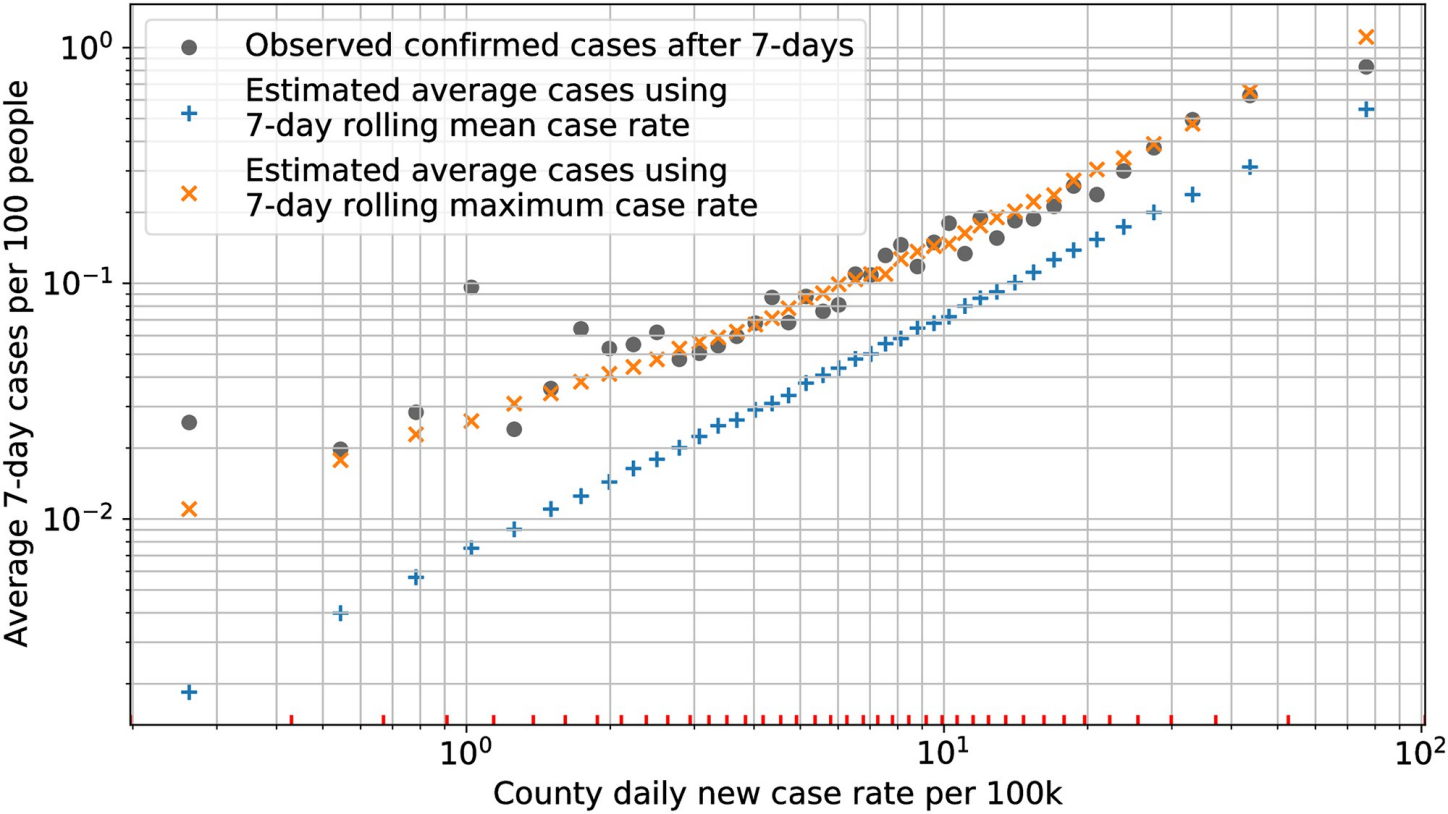
Average 7-day cumulative incidence. Comparison of average confirmed cases in nursing homes prior to and including the week of case introduction, with the model estimates of average cases in 1 week. The red ticks on the x-axis mark the 7-day mean case-rate boundaries of each category bin.

Fig 3 shows the comparison of the average number of confirmed cases in nursing homes within each bin, with the average estimate of cases from model simulations for each bin. Within each bin, the model estimates are generated with two different case rates—geometric mean of the 7-day mean case rate, and geometric mean of the 7-day maximum case rate. The observed data and both the model estimates are plotted on the same abscissa marking the geometric mean of the 7-day mean case rate for each bin.

Similar to the previous comparison, we observe from the figure that the average confirmed cases are higher than the estimated cases when using the 7-day mean case rate, and match closely with the estimate based on 7-day maximum case rate. The same factors as described previously are likely to contribute to this variation. Additionally, our assumptions about the daily interactions and the SAR parameters in nursing homes could also contribute to the differences.

#### Distribution of 7-day cumulative incidence

Finally, we compared the distribution of cases in nursing homes with the distribution of cases generated from model simulations. Given the closer correlation of observation averages and model averages for 7-day maximum case rates, this analysis was done only based on the 7-day maximum case rates.

**Fig 4:**
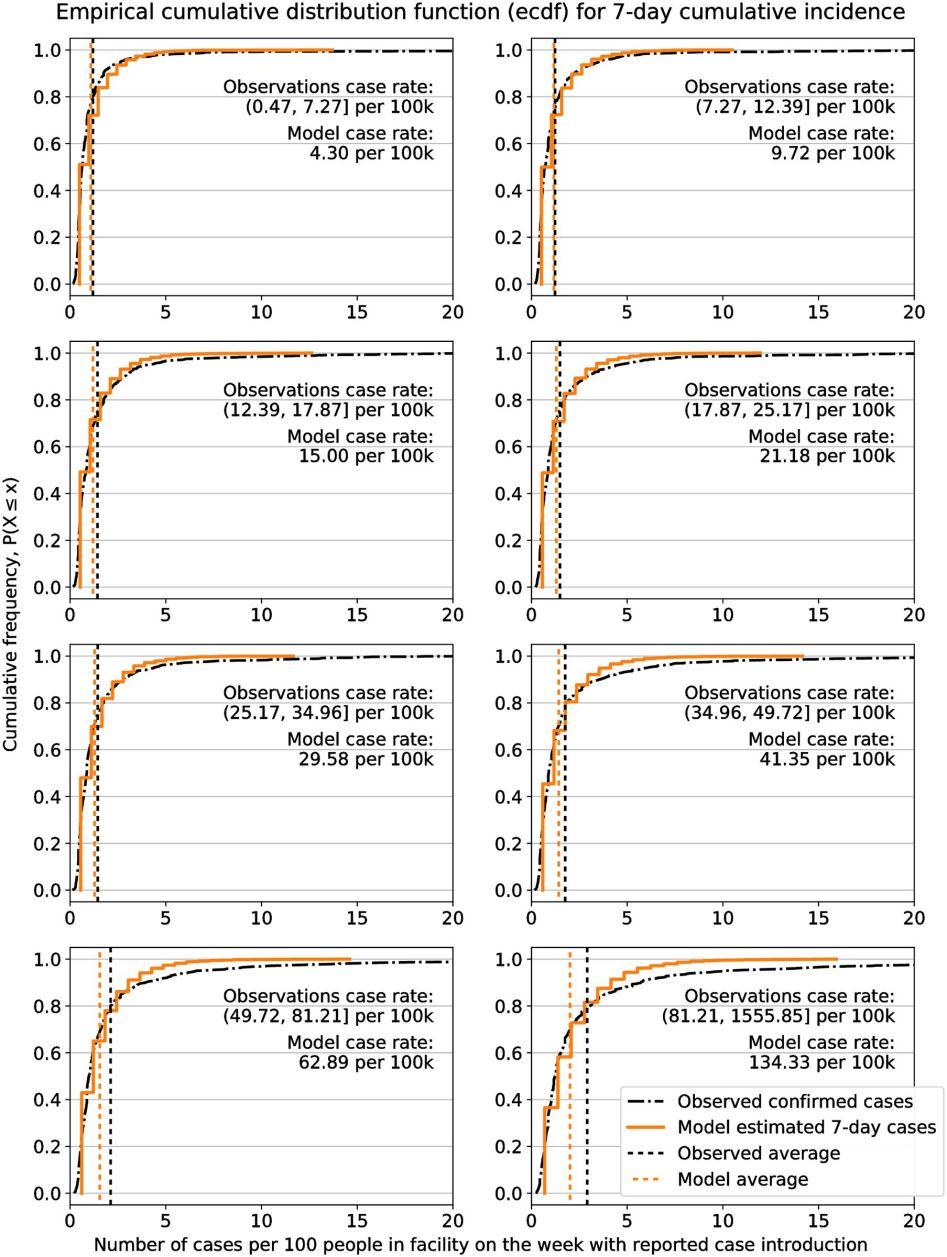
Distribution of 7-day cumulative incidence. Comparison of the distribution of cases per 100 people in nursing homes during the week of case introduction, and 7-day model simulations. Each subplot describes the distribution for facilities within one case rate category bin, and for the model based on corresponding mean case rate for that bin. Vertical dashed lines represent the average cases for the facilities and the simulations in each bin.

The comparison of the distribution of cases between observations and model estimates was done only for facilities with at least one reported case. We divided these ≈ 8000 facilities into 8 category bins by their 7-day maximum case rate, with ≈ 1000 facilities in each bin. A separate model analysis was run for each category bin using the geometric mean of 7-day maximum case rate, and average number of beds for all facilities in the bin, as described previously. The distribution of confirmed cases in each unique facility within a bin during the week of case introduction were compared with the 7-day cumulative incidence outputs from the model only for those simulations that had at least one case at the end of the 7th day. The comparison for each of the bins is shown in Fig 4. We observe a strong correlation between the distribution of cases across facilities and the distribution of cases based on model simulations. This confirms that the model presented in this study provides a good basis for estimating the distribution of cases within a workplace over a 7-day period.

A similar comparison was done for the distribution of cumulative incidence after 14 days, with similar results, and shown in S2 Appendix. Based on our comparison with observations from nursing homes, we conclude that the model provides reasonable estimates for spread of COVID-19 within facilities for short forecast periods of up to 15 days.

## Discussion

In this section, we present the key limitations of the current study, and motivations for future work.

### Limitations of the model

The current model has the following limitations -

- The model only keeps track of the first and second generations of infections. However, evidence suggests that it is possible that multiple generations of transmission events could occur within a short period. In the report for the Tianjin Office outbreak [58], the third-generation cases occurred on the 7th day post case introduction. However, for most outbreaks, we expect limited transmission events within the intended short forecast period, as evidenced by the similarities in model outputs with observations. We have included an assessment of this assumption by comparing the results with a preliminary model that includes third-generation cases in S5 Appendix. Additionally, we expect that the underestimation of cumulative incidence by ignoring third-generation cases is somewhat balanced by the overestimation due to first-generation cases being infectious for the entire forecast period. Multiple studies show a highly variable infectious period distribution ranging from 5 to 20 days depending on the viable viral load [66–69].
- We did not differentiate among the droplet, aerosol, and direct contact modes of COVID-19 transmission [70, 71]. There is evidence of long-distance COVID-19 airborne transmission [72, 73]. However, the advantage of this abstraction is that it did not require consideration of different SAR for different interactions, and allowed us to adjust SAR based on airflow and filtration. Overall, since we are focusing on outbreaks in workplaces where people are expected to be in close distance less frequently than in household settings, they are more likely to be infected with aerosol particles that travel longer distances and stay longer in the air [74–77].
- The use of air changes per hour within facilities is a simplified proxy for estimating the effects of airflow. A study on airborne transmission risk in practical settings [20] demonstrated that the location of vents also plays a critical role in aerosol transmission risk.

### Limitations of the data

- Underreporting, undertesting, and lagging of tests are common issues in reporting of COVID-19 data, especially prior to March 2020 when there was a lack of testing supplies for RT-PCR tests in the United States [78, 79]. The RT-PCR screening test of COVID-19 takes one to several days depending on the laboratory capacity [80, 81]. Additionally, it is likely that asymptomatic individuals are not getting tested. Therefore, the case rate data used in the model may not reflect the actual incidence rates.
- We did not explicitly assess asymptomatic cases in the current model. The proportion of asymptomatic cases can change the implementation of SAR distribution [82, 83]. However, this proportion is associated with numerous factors, including virus genetics [84], host factors [85, 86], generation time [32, 87], etc., and this information is not readily available in epidemiological reports of COVID-19 outbreaks.
- Detailed temporal data on outbreaks in workplaces is scarce. There have been a small number of published epidemiological reports with comprehensive information about major outbreaks, however to our knowledge most state-level public health departments in the United States do not specifically publish data for workplace outbreaks. Comprehensive data collection and publication for outbreak events in various workplaces would be highly valuable for policy makers, businesses, and researchers to facilitate disease surveillance and monitoring, and to evaluate the transmission risk in the workplace environment.

### Future work

The model presented here provides a framework to comprehensively account for the uncertainties in the spread of COVID-19, so that the number of cases can be estimated for small populations and short durations. The underlying framework can be expanded further to incorporate other capabilities.

- Workplaces like restaurants and retail stores can be modeled by removing the limitation of a static population. As a result, instead of first-generation cases only being introduced in the staff population, they can also be introduced by external visitors, for example, customers in a restaurant.
- Heterogeneous interactions can be incorporated in the contact network, for example, to model different extent of interactions within and outside different departments, or between different population groups like staff and residents in nursing homes; and to model events like meetings and meals where many people interact at the same time.
- Heterogeneity in SAR can be incorporated for different population groups, for example, by age, sex, presence of symptoms, and vaccination status, and for different modes of transmission, like droplet, airborne, or fomite.
- We modeled the long-tail behavior of SAR distribution using the Beta distribution. This choice of distribution can be evaluated as more information becomes available from contact tracing and in-depth analysis on SAR.
- Longer duration forecasts can be modeled by incorporating incubation period and time to recovery for exposed and infectious individuals. This would enable modeling third and subsequent generations of transmission, and intervention measures like quarantining and isolation during the forecast duration.
- SAR and other parameters can be changed to model the impact of emerging COVID-19 variants [88, 89], and other airborne diseases.

## Conclusions

We presented a stochastic contact network model to forecast the spread of COVID-19 over short duration of ≤15 days, and for small populations within workplaces. The model incorporated cases arriving at a workplace from the local community, and the secondary transmission between the infectious and susceptible populations in the facility. Introduction of newly infected individuals was modeled probabilistically through a Bernoulli process with the success rate as the local daily new case rate. Transmission within the facility was determined by probabilistic realizations of both the number of secondary contacts for each infectious individual, and their corresponding secondary attack rates (SAR). By running multiple simulations with realizations from the probability distributions of these model parameters, we obtained a distribution of predicted cumulative incidence within a facility over the forecast period.

The short forecast duration was chosen in this study because we expect that interventions are often taken in workplaces soon after identification of cases, and as a result, longer duration forecasts may have higher variability and thus be less informative for policy and decision makers.

The model presented here was validated with respect to two different datasets. In the first part, we evaluated whether observations of cumulative incidence from 7 real-world outbreaks in workplaces could be estimated by the model. For all outbreaks examined, the observations lied within the range of model simulations. In the second part, we compared the model with weekly case reports from over 8000 nursing homes in the United States. We observed a strong correlation between the observed probability of first-generation individuals being introduced in the facility, and the average cumulative incidence after 7 days. We also compared the distribution of cumulative incidence after 7 days, and found a strong correlation with the distributions from model simulations.

We demonstrated that the contact network model presented in this study is a simplified but reasonable representation of the cumulative incidence of COVID-19 within workplaces for up to 15 days and populations as small as 50 individuals. We believe that it provides a consistent framework to account for uncertainties in disease transmission, serve as a data-driven tool for occupational safety, and can be expanded to model outbreaks for emerging COVID-19 variants and other airborne diseases.

The model described here is implemented in a web application, available at covid19.oneconcern.com. The open source model code implemented in python:3 is available at github.com/abhineetgupta/covid-workplace-risk.

## Supporting information

S1 Appendix Individual outbreak reports from workplaces for model validation. (PDF)

S2 Appendix Nursing homes dataset for model validation. (PDF)

S3 Appendix Relation of secondary attack rate with respect to source emissions and inhaled virions. (PDF)

S4 Appendix Model convergence. (PDF)

S5 Appendix Comparison with a model with additional generation. (PDF)
