# Supplementary material for: A stochastic contact network model for assessing outbreak risk of COVID-19 in workplaces
Source: PLoS One. 2022 Jan 14;17(1):e0262316. doi: 10.1371/journal.pone.0262316 (PMC8759694; doi:10.1371/journal.pone.0262316)
Supplement: S1 Appendix — (PDF) [file pone.0262316.s001.pdf]

S1 Appendix - Individual Outbreak Reports from Workplaces for Model Validation

In this appendix, we provide detailed information regarding validation of the stochastic models with data from real-world outbreaks. We collected epidemiological reports on COVID-19 outbreaks in workplace-like situations as shown in Table 1. These cases were collected through literature and media review and selected based on the following criteria -

- The outbreak must have occurred in a workplace-like setting, and excludes facilities with visitors, such as schools and restaurants.
- Cumulative incidence must be reported or inferable.
- The case introduction date must be reported or inferable.
- The report includes information about the number of people involved, location of the outbreak, detailed timeline of the events, and methods of diagnosis.

Parameter Selection

We assumed a set of baseline parameters for most of the case studies based on the most common settings in workplaces. Additionally, since no information was available about the environmental settings in these outbreaks, instead of selecting a single parameter value, we selected a range based on expert judgement. The comparisons are then presented for both the lower and the upper bound estimates using the values from the range.

Table 1. Outbreak Reports for Workplace Outbreaks. Model parameters associated with outbreak reports used for validation

**Outbreak Name**	e	p_{case} (per 100k)	$\bar{c}$	λ_a (ACH)	s_v (dB)	s_p (%)	n_{days}	p_{remain}
1. Tianjin Office [1]	906	1	4-10	1.5-4	65	5-25	10	0.4
2. Korean Call Center [2]	216	1	4-10	1.5-4	70	55-66	14	0.4
3. San Diego VA Office [3]	100	1	4-10	1.5-4	65	5-40	8	0.4
4. Singapore Conference [4]	111	1	6-12	1.5-4	70	25-40	3	0
5a. South Dakota Meat Processing Plant (First Shift) [5]	1744	1	4-10	1-11	70	5-20	14	0.4
5b. South Dakota Meat Processing Plant (Second Shift) [5]	1459	1	4-10	1-11	70	5-20	14	0.4
6. Henan Expressway [6]	103	1	2-4	N.A.	N.A.	N.A.	13	0.4
7a. Major League Baseball Team with Social Distancing and Mask Protocols [7]	68	60	4-8	1.5-4	65	5-25	10	0.4
7b. Major League Baseball Team without Social Distancing and Mask Protocols [7]	68	60	4-10	1.5-4	65	5-25	10	0.4

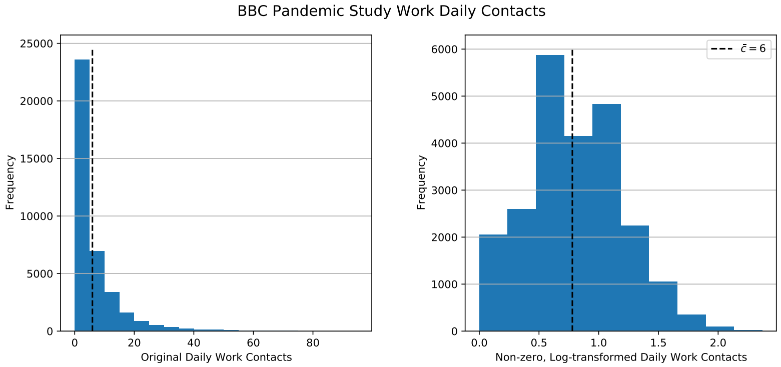

Fig 1. Histogram of daily work contacts in the BBC pandemic study left: original data; right: log 10-transformed non-zero data

Based on the BBC pandemic study, the average daily contacts are $\bar{c} = 6$. After transforming the data to the Gaussian distribution as shown in Fig 1, we selected average contacts as the range corresponding to the 30th and 70th percentiles, hence $\bar{c} = [4, 10]$. The probability of contacts that remain the next day is $p_{\text{remain}} = 60\,\%$. In some cases, we adjusted the average daily contacts based on specific conditions as described in their respective sections. Daily contacts differences due to change of cultural, behavior, and public health policy factors across different countries were not considered.

We obtained design airflow, or air changes per hour (ACH) in workplaces from data published on ventilation system supplier websites and technical construction websites [8–11]. We estimated the actual airflow in the range of 1.5 ACH–4 ACH, by halving the design ACH in these reports based on a study that concluded that the maximum measured air change rate is about one half of the design capacity [12]. We assumed that there is no filtration in these workplaces.

Normal human speech is generally in the range of 60 dB–70 dB [13–15], hence we assumed a speaking volume of $s_v = 65\,\text{dB}$. Based on a review of 17 companies, a study concluded that on average 15 % time is spent by employees in meetings, which increases to 40 % for senior executives [16]. As a result, we assumed the average speaking percentage range as $s_p = [5\,\%, 25\,\%]$.

For most of the case studies, we used the average local daily new case rate within the week prior to the first case introduction in their respective locations. When local case rates were less than 1 per 100k, we used $p_{\text{case}} = 1$ per 100k as the lowest value. The final parameters used for each outbreak are listed in Table 1.

Results of Comparisons with Outbreak Reports from Workplaces

Tianjin Office Outbreak

An outbreak occurred among railroad staff members in an administrative office building in the Tianjin province in China starting on 2020-01-15, with 17 total cases, including both workplace and household contacts. We only modeled workplace transmission from 2020-01-15 to 2020-01-24, with total 7 workplace cases, including 2 third-generation cases. The total number of employees in the office was $e = 906$ based on the report, although there was no information about how many employees were in the building during this period. The index patient showed clinical signs after visiting Wuhan with a colleague. The disease was transmitted to other staff via daily interactions at work, meetings, and traveling together. We assumed this office setting is representative of a

Table 2. Observations and Model Estimates for Workplace Outbreak Reports. Observations and model outputs of cumulative incidence for outbreaks with $n_{\mathrm{sim}} = 5000$ for each model. I_{obs} refers to the observed cumulative incidences; %-ile refers to the percentiles where the observations fall within the predicted range and N.A. indicates the observation is outside the range; IQT refers to the interquartile range, or the 25th to 75th percentile range of the model output.

Outbreak Name	I_{obs}	Lower Bound Model				Upper Bound Model			
		%-ile	range	IQT	median	%-ile	range	IQT	median
1. Tianjin Office	7	99.9	1 - 9	1 - 2	1	58.9	1- 55	3 - 10	5
2. Korean Call Center	76	N.A.	1 - 34	2 - 8	4	97.9	1 - 135	10 - 38	22
3. San Diego VA Office	5	99.9	1 - 5	1 - 1	1	45	1 - 55	3 - 11	6
4. Singapore Conference	7	99	1 - 12	1 - 2	1	74	1 - 55	2 - 8	4
5a. SD Meat Processing (First Shift)	32	N.A.	1 - 7	1 - 2	1	88.9	1 - 102	5 - 21	12
5b. SD Meat Processing (Second Shift)	6	99.9	1 - 7	1 - 2	1	26.7	1 - 108	5 - 21	11
6. Henan Expressway	6	60	1 - 30	2 - 8	5	36	1 - 51	4 -14	8
7a. Major League Baseball Team with Social Distancing and Mask Protocols	20	N.A.	1 - 8	1 - 2	1	99.8	1 - 28	2 - 6	4
7b. Major League Baseball Team without Social Distancing and Mask Protocols	20	N.A.	1 - 9	1 - 2	1	95.2	1 - 43	3 -10	6

common office and hence used our baseline parameters. Employees did not take any precautions, hence we assumed that no one wore masks, and $m_e = 0$.

The distribution of cumulative incidence for both the lower bound and the upper bound models is shown in Fig 2. Since only those simulations with at least one case on the first day were considered, the cumulative incidence ≥ 1. For the lower bound model, simulated cumulative incidence was 1–9, and for the upper bound was 1–55 (Table 2). Hence, the observation lies within the range of model simulations.

We also compared temporal incidences in this study, as shown in Fig 3. The cumulative incidence on each day also lies within the range of the simulations.

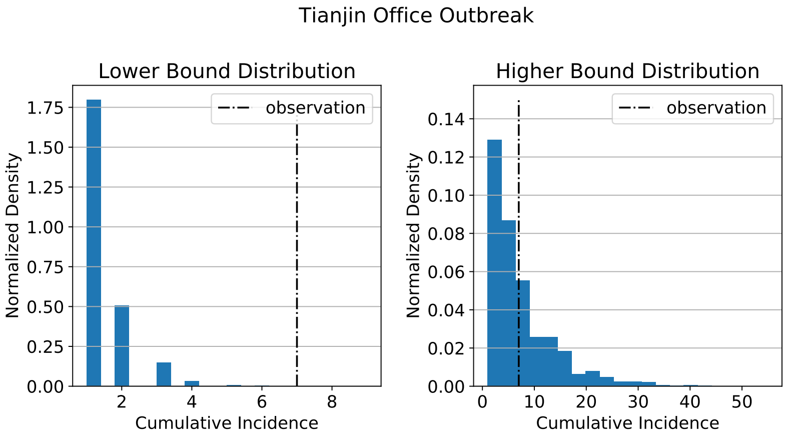

Fig 2. Comparison of Model Simulation and Observation in Tianjin Office Outbreak. Distribution of cumulative incidence 10 days post case introduction from the lower bound model (left) and the upper bound model (right) for the Tianjin Office outbreak, compared with the observation

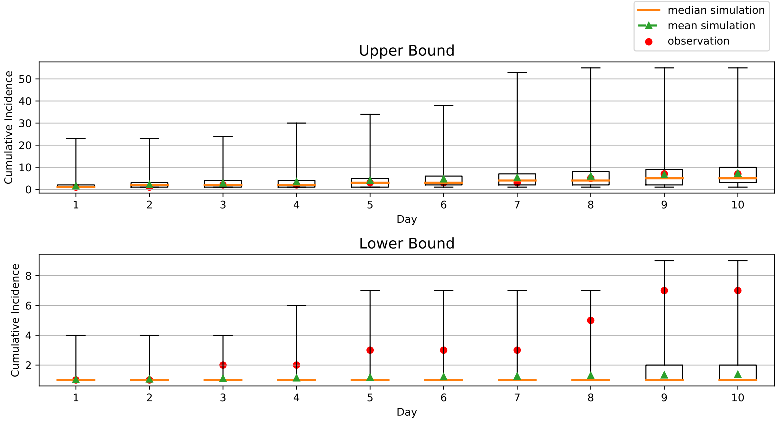

Fig 3. Temporal Dynamics of the Tianjin Office Outbreak. Boxes represent the interquartile range, while whiskers represent the entire range of estimates from the model simulations.

Korean Call Center Outbreak

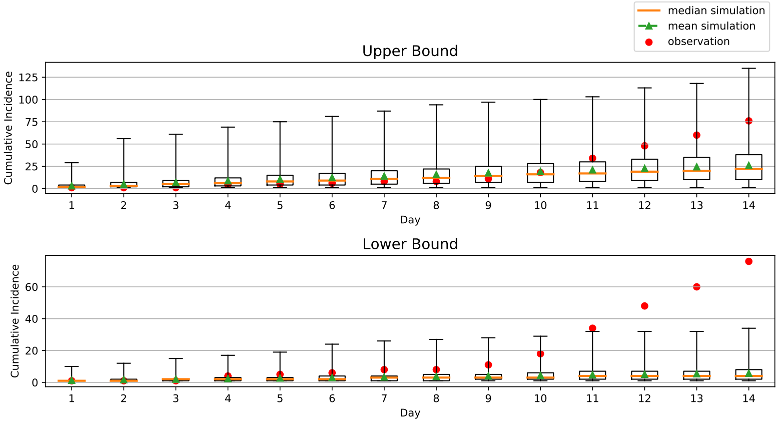

Fig 4. Temporal Dynamics of the Korean Call Center Outbreak. Boxes represent the interquartile range, while whiskers represent the range of estimates from the model simulations.

There was an outbreak with 97 confirmed cases in a call center on the 11th floor of a 19-story building in the urban area of Seoul from 2020-02-25 to 2020-03-09 [2]. Of the 97 confirmed cases, 94 worked at the call center on the 11th floor, with the remainder worked on other floors. Therefore, we limited the model estimation to the $e = 216$ employees on the 11th floor. We modeled the first 14 days prior to the closure of the building. There were a total of 76 cases within the estimation window. We expect more talking, and relatively higher speaking volume in typical call centers compared with a common workplace [17], hence we assumed that $s_p = [55\,\%, 66\,\%]$, and $s_v = 70\,\text{dB}$. The corresponding average SAR for this speaking activity was $\overline{\text{SAR}} = [8.88\,\%, 21.54\,\%]$.

The observed cumulative incidence lies within the simulation ranges for the upper bound model (range = 1–135, median = 22). The temporal observations are also within the temporal simulation range of the model, as shown in Fig 4. However, the observations exceeded the lower bound model starting on the 11th day. This could be

because the lower bounds parameters are not as representative of the call center as the upper bound parameters. Moreover, the index case of the entire outbreak was from 10th floor (not included in the model) and occurred 3 days prior to the first case introduction on the 11th floor, hence there could be more case introductions than estimated based on our selected parameters. Another possibility is that in a call center with high occupancy, and constant talking, heightened transmission risk is likely to lead to more third-generation cases within 14 days.

San Diego VA Office Outbreak

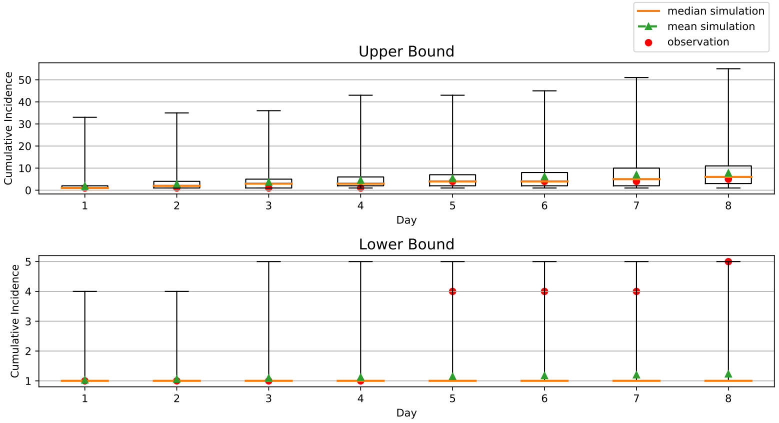

Fig 5. Temporal Dynamics of the San Diego VA Office Outbreak. Boxes represent the interquartile range, while whiskers represent the range of of estimates from the model simulations.

An outbreak at a VA counseling office in San Diego in United States starting on 2020-03-14 resulted in 32 cases [3]. The report stated that one-third of the total employees got sick, hence we assumed that there were $e = 100$ employees in the office. We assumed a modeling period of 8 days since the staff were asked to work remotely 8 days post first case introduction. Although there were visitors going to the counseling center, visitor information or contact tracing were not reported, and we assumed the transmissions were mainly via within-workplace interactions between employees. Since this was a counseling center, we assumed a higher upper bound percentage of speaking $s_p = [5\,\%, 40\,\%]$. The observed cumulative incidence aligns with estimates from both the upper and lower bound models. The temporal cumulative incidences are within the range of both the upper bound and the lower bound models as shown in Fig 5.

Singapore Conference Outbreak

An outbreak resulting in 7 cases was associated with a company-wide conference held in Singapore from 2020-01-20 to 2020-01-22 [4]. There were at least 111 participants from 19 different countries. The presumed first-generation case was among a group of 17 people from China, with at least one from Wuhan. The report illustrated the agenda for this conference, including multiple instances of dining and group sessions. Therefore, we assumed higher average daily contacts corresponding to the mean and the 85th percentile from the BBC study $\bar{c} = [6, 12]$. We also assumed higher speaking percentage $s_p = [25\,\%, 40\,\%]$. The observational value 3 days post first case introduction lies within the range of model simulations as shown in Table 2.

South Dakota Meat Processing Plant Outbreak

An outbreak resulting in 929 confirmed cases among 3635 employees occurred in a meat processing facility in South Dakota in United States from 2020-03-16 to 2020-04-25 [5]. The first case was identified in the first shift on 2020-03-16. We updated the airflow (ACH) based on general recommendations for food processing facilities $\lambda_a = [1, 11]$ [9,18]. Additionally, we assumed that there was a lower than the baseline level of speaking activity $s_p = [5\,\%, 20\,\%]$ in the meat processing plant compared with common offices, as employees were expected to have fewer meetings than in an office. There were three shifts in the facility - the first two shifts were regular operations, and the third shift was responsible for cleaning. We considered the first two main shifts independently for the model estimations. The first two weeks were used as the estimation window, because intervention measures took place in the third week. We assumed a baseline level of average daily contacts, but an increased speaking volume $s_v = 70\,\mathrm{dB}$ to account for more ambient noise in a factory. The observational cumulative incidences for both shifts lie within the range of upper bound model simulations.

Henan Expressway Service Area Outbreak

An outbreak resulting in 6 cases occurred at a highway service area in Henan province in China. The area consisted of restaurants, shopping, car service, and lounging areas [6]. The index case was an employee who was infected by a visitor from Wuhan who stopped at the service area on 2020-01-22. The employee started to have symptoms on 2020-02-04. During 13 days post first case introduction, a total of 6 employees were infected through interactions at work, sharing living space, and having meals together. The index case was diagnosed and removed from the office, but the interaction within the living space still remained for 4 days. Since employees shared living space, the secondary transmission was similar to households, hence we used the contact data corresponding to households from the BBC study $\overline{c} = [2, 4]$, and the household $\overline{\mathrm{SAR}} = 20\,\%$ based on a meta-analysis [19]. Overall, the observation lies within the ranges of both lower and upper bound model simulations.

Major League Baseball Team Outbreak

An outbreak resulting in 21 cases started at the beginning of the 2020 baseball season during late July and August (dates not disclosed), involving Team A travelling to location C and location B to play with two other teams [7]. Since 20 cases were from Team A over a 10-day period, we focused the simulation only on this team. We used the case rate in the county of Philadelphia at the time of $p_{\mathrm{case}} = 60$ per 100k. While social distancing and mask protocols were implemented within the club facility, the compliance during off-game time and in private were not reported. Therefore we tested two different scenarios - in the first scenario, we assumed some compliance, and hence reduced average contacts $\overline{c} = [4, 8]$, and implemented mask effectiveness $m_e = 0.44$; and in the second scenario, we assumed no masks and baseline contacts. For the former scenario, the observation lies within the range of upper bound model simulations, but exceed the lower bound simulations. Similarly, the observation for the latter scenario lies only within the range of the upper bound model simulations. This suggests that the upper bound parameters may be better representative of the conditions in this outbreak.

References

1. Zhang Y, Su X, Chen W, Fei CN, Guo LR, Wu XL, et al. Epidemiological investigation on a cluster epidemic of COVID-19 in a collective workplace in

Tianjin. Zhonghua Liu Xing Bing Xue Za Zhi = Zhonghua Liuxingbingxue Zazhi. 2020;41(5):648–652. doi:10.3760/cma.j.cn112338-20200219-00121.

2. Park SY, Kim YM, Yi S, Lee S, Na BJ, Kim CB, et al. Coronavirus Disease Outbreak in Call Center, South Korea. Emerging Infectious Diseases. 2020;26(8):1666–1670. doi:10.3201/eid2608.201274.

3. Castellano J. How A COVID-19 Outbreak Unfolded At A San Diego VA Office; 2020. Available from: https://www.kpbs.org/news/2020/may/21/how-covid-19-outbreak-unfolded-san-diego-va-office/.

4. Pung R, Chiew CJ, Young BE, Chin S, Chen MIC, Clapham HE, et al. Investigation of three clusters of COVID-19 in Singapore: implications for surveillance and response measures. The Lancet. 2020;395(10229):1039–1046. doi:10.1016/S0140-6736(20)30528-6.

5. Steinberg J, Kennedy ED, Basler C, Grant MP, Jacobs JR, Ortbahn D, et al. COVID-19 Outbreak Among Employees at a Meat Processing Facility - South Dakota, March-April 2020. MMWR Morbidity and Mortality Weekly Report. 2020;69(31):1015–1019. doi:10.15585/mmwr.mm6931a2.

6. Pang Q, Li P, Li T, Li H. Investigation on a cluster epidemic of corona virus dieases 2019 (COVID-19) in expressway service area. Anhui J Prev Med. 2020;26(2):130–132. doi:10.19837/j.cnki.ahyf.2020.02.013.

7. Murray MT, Riggs MA, Engelthaler DM, Johnson C, Watkins S, Longenberger A, et al. Mitigating a COVID-19 Outbreak Among Major League Baseball Players. MMWR Morbidity and Mortality Weekly Report. 2020;69(42):1542–1546. doi:10.15585/mmwr.mm6942a4.

8. Moffitt Natural Ventilation Solutions. Air Changes Per Hour; 2019. Available from: https://www.moffittcorp.com/air-changes-per-hour/.

9. Lakeair. Air Changes Per Hour; 2020. Available from: https://www.lakeair.com/air-changes-per-hour/.

10. Engineering Toolbox. Air Change Rates in Typical Rooms and Buildings; 2005. Available from: https://www.engineeringtoolbox.com/air-change-rate-room-d_867.html.

11. Sheriff RE. Building Ventilation – The Proper Air Changes Per Hour (ACH); 2020. Available from: https://www.atlenv.com/building-ventilation-the-proper-air-changes-per-hour-ach.

12. Dols W, Persily AK. A Study of Ventilation Measurement in an Office Building. American Society for Testing and Materials, Special Technical Publication. 1995;.

13. American Academy of Audiology. Level of Noise in decibels (dB). American Academy of Audiology; 2010. Available from: https://audiology-web.s3.amazonaws.com/migrated/NoiseChart_Poster-%208.5x11.pdf_5399b289427535.32730330.pdf.

14. Safety YEH. Decibel Level Comparison Chart. Yale Envrionment Health & Safety; 2021. Available from: https://ehs.yale.edu/sites/default/files/files/decibel-level-chart.pdf.

15. Center for Hearing and Communication. Common environmental noise levels; 2021. Available from: https://chchearing.org/noise/common-environmental-noise-levels/.

16. Mizumoto K, Kagaya K, Chowell G. Effect of a wet market on coronavirus disease (COVID-19) transmission dynamics in China, 2019-2020. International Journal of Infectious Diseases. 2020;97:96–101. doi:10.1016/j.ijid.2020.05.091.

17. Adkins C. How Many Calls Should an Outbound Agent Make; 2020. Available from: https://www.callcentrehelper.com/how-many-calls-should-an-outbound-agent-make-57157.htm.

18. Concepts and Designs Incorporated. Food Processing Plants; 2009. Available from: http://www.cdihvac.com/portal/filelist.aspx?DownloadId=235.

19. Koh WC, Naing L, Chaw L, Rosledzana MA, Alikhan MF, Jamaludin SA, et al. What do we know about SARS-CoV-2 transmission? A systematic review and meta-analysis of the secondary attack rate and associated risk factors. PLOS ONE. 2020;15(10):e0240205. doi:10.1371/journal.pone.0240205.
