# Supplementary material for: A stochastic contact network model for assessing outbreak risk of COVID-19 in workplaces
Source: PLoS One. 2022 Jan 14;17(1):e0262316. doi: 10.1371/journal.pone.0262316 (PMC8759694; doi:10.1371/journal.pone.0262316)
Supplement: S2 Appendix — (PDF) [file pone.0262316.s002.pdf]

S2 Appendix - Nursing Homes Dataset for Model Validation

The Division of Nursing Homes; the Quality, Safety, and Oversight Group; and the Center for Clinical Standards and Quality at the Centers for Medicare and Medicaid Services have been compiling and publishing COVID-19 data in nursing homes or long-term care facilities across the US since May 2020 [1]. Here, we describe the relevant data attributes, data processing, and underlying assumptions for using this dataset for validating our model.

Data description

The nursing home dataset includes confirmed COVID-19 cases in the facilities reported on a weekly basis since 2020-05-24. For this study, the dataset was downloaded on 2021-03-03, and includes the dataset last updated on 2021-02-25. We removed the nursing homes that reported confirmed cases on 2020-05-17 because in the dataset the cases reported on this day is not the weekly report but cumulative of all previous cases and thus the date of first case introduction cannot be ascertained.

Within the scope of this study, the following columns in the dataset were used, where each row represents data from one facility reported for one week -

1. *Week Ending* - Used to determine reporting date for facility
2. *Federal Provider Number* - Used as unique identifier for facility
3. *County, Provider State* - Used to identify facility county, which was used to determine county case rate for a given week
4. *Number of All Beds* - Used as an approximation for the number of residents in the facility
5. *Residents Weekly Confirmed COVID-19, Staff Weekly Confirmed COVID-19* - The sum of these columns for each row was used as the total confirmed cases in a facility on a given week.

We used the number of beds as an approximation for the number of residents. Additionally, a report by the National Center for Health Statistics concluded that there were approximately 90 full-time staff (including administrative, medical, and therapeutic staff) per 100 residents based on a survey of nursing homes across the US [2]. Hence, we approximated the staff population as 90% of the number of beds.

For each facility and each week, the county-level daily new cases for that week were determined from the New York Times dataset [3]. The population data for each county was obtained from the U.S. Census Bureau [4]. Two types of weekly case rates were computed for each facility -

- **7-day mean case rate** - the average of the daily new cases per 100,000 population during the previous 7-days from the reporting date of the facility, and
- **7-day maximum case rate** - the maximum of the daily new cases per 100,000 population during the previous 7-days from the reporting date of the facility.

For each facility, the weekly data was classified into the following categories -

1. **Case introduction week** - the first week when at least one confirmed case was reported in the facility. The case introduction week is unique for each facility. It is possible for a facility to have no case introduction if no confirmed cases have been reported in the studied period.
2. **Pre-introduction weeks** - all weeks between 2020-05-24 until, but not including the introduction week, when zero confirmed cases were reported in the facility. The number of pre-introduction weeks are variable for each facility. For example, a facility with an introduction-week date of 2020-07-19 has 7 pre-introduction weeks, starting from 2020-05-24. Since no cases were reported in these weeks, we assume that zero cases were introduced within the last 7 days from each of the reporting dates of these weeks. For facilities with no reported cases, all weeks within the analyzed period are considered pre-introduction weeks.
3. **First post-introduction week** - the first week after the introduction week for each facility. The subsequent weeks after the first post-introduction week were excluded from the dataset since it could not be determined whether the source of infection was new introductions or in-facility transmissions after the first case introduction. The first post-introduction week was used for comparing against 14-day model estimates. Facilities with no introduction week also have no post-introduction weeks.

As a result of this data preprocessing, there are 8423 unique facilities in 2463 unique counties in the dataset for the analyzed period, totalling 89,273 data entries. On average, the facilities had 84.47 beds. 8235 facilities (97.7%) reported at least one confirmed case in this period. Of these, most of the facilities reported their first confirmed case in earlier months of the dataset - 4829 (58.6%) reporting their first case by the end of July 2020, and 6883 (83.6%) by the end of September 2020.

Parameter Selection

We assumed that the number of daily interactions in nursing homes is equivalent to those in workplaces, hence $\overline{c} = 6$ average contacts per person per day. Similarly, we assumed that the average SAR in nursing homes is $\overline{\mathrm{SAR}} = 5.1\,\%$. This SAR value closely matches the observation of 5 % in a survey of 12 nursing homes in France [5].

Model Modification for Nursing Homes

Nursing homes are comprised of resident and staff population. Generally, the resident population remains within the facility, while the staff population attends the facility each day similar to a workplace. We incorporated this separation in the facility population by modifying our original model.

Compared to workplaces where any member of the susceptible population can be a first-generation case arriving at the facility, for nursing homes, we assumed that only the staff population can be first-generation cases. We made this simplification since the visitation information was unknown and and no contact-tracing data was reported. Once first-generation cases have been introduced, we assumed that subsequent transmission within the facility occurred in a homogeneous population, and we did not make any further distinction in the two population groups. In reality, we expect some differences between staff-to-staff, staff-to-resident, and resident-to-resident interactions, however these differences were not modeled due to a lack of interaction data.

There were two different sets of models for the comparisons described below, that we have classified into two stages. In **stage-1**, the model was run for a 7-day period. The

average estimates from these simulations were compared against the average observed cases in all facilities in their pre-introduction and introduction weeks. In **stage-2**, the model was run for a total of 14 days, but for only those simulations that had at least one case at the end of the 7th day. These estimates were compared with the distribution of cases in the introduction-week and the first post-introduction week of the facilities. Each run of the model included $n_{\text{sim}} \geq 10000$ simulations to ensure convergence of the outputs.

Results

In addition to the results included in the main text, in this section, we have included a comparison of the distribution of cases in nursing homes with the distribution of cases generated from model simulations on the week following the first reported case. All parameters for this comparison are the same as that for the 7-day comparison. Hence, this represents the comparison of cumulative incidence after 14 days, and is shown in Fig 1. We observe a strong correlation between the distribution of cases across facilities and the distribution of cases based on model simulations. This confirms that the model presented in this study provides a good basis for estimating the distribution of cases within a workplace over a 14-day period.

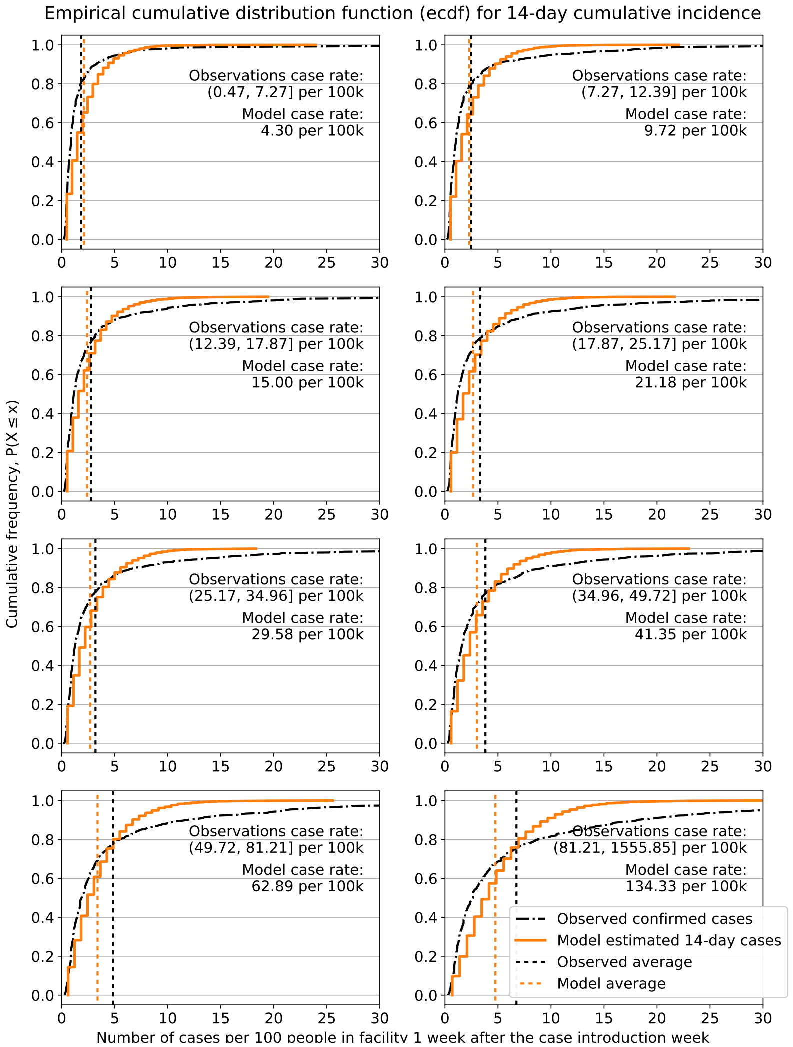

Fig 1. Distribution of 14-day Cumulative Incidence. Comparison of the distribution of cases per 100 people in nursing homes following the week of case introduction, and 14-day model simulations. Each subplot describes the distribution for facilities within one case rate category bin, and for the model based on corresponding mean case rate for that bin. Vertical dashed lines represent the average cases for the facilities and the simulations in each bin.

References

1. Centers for Medicare and Medicaid Services - Division of Nursing Homes/Quality, Safety, and Oversight Group/Center for Clinical Standards and Quality. COVID-19 Nursing Home Dataset; 2021. Available from: `https://data.cms.gov/Special-Programs-Initiatives-COVID-19-Nursing-Home/COVID-19-Nursing-Home-Dataset/s2uc-8wxp`.

2. Gabrel CS. An Overview of Nursing Home Facilities: Data from the 1997 National Nursing Home Survey. Advance Data. 2000;(311):12.

3. Almukhtar S, Aufrichtig A, Barnard A, Bloch M, Cai W, Calderone J, et al.. Coronavirus (Covid-19) Data in the United States; 2021. Available from:

https://github.com/nytimes/covid-19-data.

4. United States Census Bereau. 2019 U.S. County Population; 2020. Available from: https://www2.census.gov/programs-surveys/popest/datasets/2010-2019/counties/asrh/.

5. Reyné B, Selinger C, Sofonea MT, Miot S, Pisoni A, Tuaillon E, et al. Wearing masks and establishing COVID-19 areas reduces secondary attack risk in nursing homes. medRxiv. 2020; p. 2020.11.27.20239913. doi:10.1101/2020.11.27.20239913.
