# Supplementary material for: A stochastic contact network model for assessing outbreak risk of COVID-19 in workplaces
Source: PLoS One. 2022 Jan 14;17(1):e0262316. doi: 10.1371/journal.pone.0262316 (PMC8759694; doi:10.1371/journal.pone.0262316)
Supplement: S3 Appendix — (PDF) [file pone.0262316.s003.pdf]

S3 Appendix - Relation of Secondary Attack Rate with respect to Source Emissions and Inhaled Virions

In this appendix, we describe the assumptions that we used to simplify the relationship between secondary attack rate (SAR) with respect to source emissions and inhaled virions.

In one study [1], SAR is defined for airborne transmission of COVID-19 with respect to inhaled virions as -

$$\mathrm{SAR} = 1 - \exp^{\frac{-N}{N_0}} \tag{1}$$

where N is the total number of virions breathed in, and N_0 is the threshold of virions required to infect a susceptible individual, or the infectivity threshold.

In the same study, the number of inhaled virions N is estimated as -

$$N = BTC_{eq}\left(1 - \frac{1}{\lambda T}\left(1 - \exp^{-\lambda T}\right)\right)$$

and

$$C_{eq} = \frac{S}{\lambda V} \tag{2}$$

where B is breathing rate, T is time exposed, C_{eq} is the concentration of virions in a steady state, λ is the decay rate of virions, S is the virion emission at the source, and V is the volume of the room.

We make the following simplifying assumptions for estimating average $\overline{\mathrm{SAR}}$ -

- We assume independent transmission on each day from an infectious individual to susceptible contacts, hence $T = 1$ day and is constant.
- The term $\left(1 - \frac{1}{\lambda T}\left(1 - \exp^{-\lambda T}\right)\right)$ in equation 2 accounts for virion decay over short periods of time. For transmission over longer duration like a day, the term may be ignored since $\lim_{T\to\infty}\left(1 - \frac{1}{\lambda T}\left(1 - \exp^{-\lambda T}\right)\right) \to 1$.
- We assume the breathing rate B to be constant. The breathing rate changes with activities like during exercise, which is not expected to occur commonly within a workplace. Breathing rate also changes based on whether masks are worn, for which we introduce an additional parameter m_e that combines the contribution of masks from both source emission rate and breathing rate.
- For a general facility, we ignore the volume (V) since people who are closer than 6 ft from the infected person are most likely to get infected [2], hence the room volume is not expected to contribute significantly to changes in SAR.
- While the infectivity threshold varies by individual, for the assessment of average $\overline{\mathrm{SAR}}$, the average infectivity threshold, N_0 is assumed as constant. For simplification, we replace $\frac{N}{N_0}$ with N' in the equations below.

Then, equation 2 is simplified to -

$$N' \propto \frac{S(1 - m_e)}{\lambda}$$

$$\implies \frac{N'_i}{N'_j} = \frac{S_i(1 - m_{e_i})}{\lambda_i}\frac{\lambda_j}{S_j(1 - m_{e_j})}$$

and

$$\overline{\mathrm{SAR}} = 1 - \exp(-N') \tag{3}$$

where subscripts i and j represent any two sets of parameters, and $m_e \in [0, 1]$ is the mask effectiveness. The source emission rate S is a function of speaking percentage and volume. The decay rate λ is a function of airflow and filtration.

References

1. Prentiss M, Chu A, Berggren KK. Superspreading Events Without Superspreaders: Using High Attack Rate Events to Estimate Nº for Airborne Transmission of COVID-19. medRxiv. 2020; p. 2020.10.21.20216895. doi:10.1101/2020.10.21.20216895.

2. Centers for Disease Control and Prevention. COVID-19 and Your Health; 2020. Available from: `https://www.cdc.gov/coronavirus/2019-ncov/prevent-getting-sick/how-covid-spreads.html`.
