# Supplementary material for: A stochastic contact network model for assessing outbreak risk of COVID-19 in workplaces
Source: PLoS One. 2022 Jan 14;17(1):e0262316. doi: 10.1371/journal.pone.0262316 (PMC8759694; doi:10.1371/journal.pone.0262316)
Supplement: S4 Appendix — (PDF) [file pone.0262316.s004.pdf]

S4 Appendix - Model Convergence

Model Convergence

In this appendix, we describe our analysis for evaluating the number of simulations needed to reach model convergence. Fig 1 shows the the average cumulative incidence for a given number of simulations. The experiments were repeated 1000 times for each set of simulations to obtain the variance. The following parameters were used - $e = 100$, $p_{\text{case}} = 10$ in 100,000, $\bar{c} = 6$, $\overline{\text{SAR}} = 5.1\%$, $n_{\text{days}} = 15$, $p_{\text{remain}} = 0.4$. As expected, the variance of the average cumulative incidence decreases with increasing simulations. For number of simulations ≥ 1000, the variance is sufficiently small.

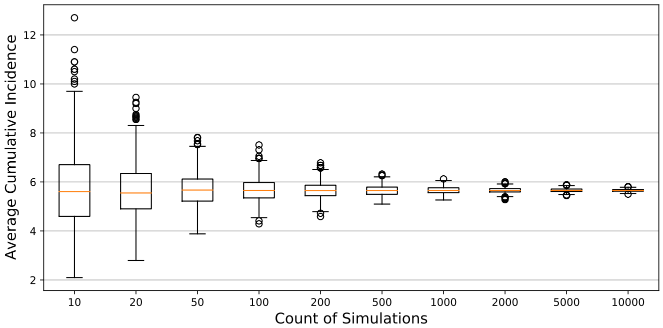

Fig 1. Boxplot of average cumulative incidence for different number of simulations The x-axis represents the simulation count.
