# Supplementary material for: A stochastic contact network model for assessing outbreak risk of COVID-19 in workplaces
Source: PLoS One. 2022 Jan 14;17(1):e0262316. doi: 10.1371/journal.pone.0262316 (PMC8759694; doi:10.1371/journal.pone.0262316)
Supplement: S5 Appendix — (PDF) [file pone.0262316.s005.pdf]

S5 Appendix - Comparison with A Model with Additional Generation

In this appendix, we describe a preliminary model that incorporates third generation cases. This model has not been validated and is provided here only for comparison with the original model.

Model Implementation

The third generation of cases was implemented on the basis of the original model after the calculation of first two generations on each day. On each day, we sample the incubation period, SAR, and the number of daily contacts for each second generation case. The incubation period for each case is sampled from a lognormal distribution [1]. The mean of this distribution is 5.32 and the median is 5.

Quantifying the Discrepancies

The following section describes the comparison of this preliminary model with the original model proposed in the study. We compared the model outputs (from 5000 simulations) with the observations in Tianjin Office and Korean Call Center outbreaks.

Tianjin Office Outbreak

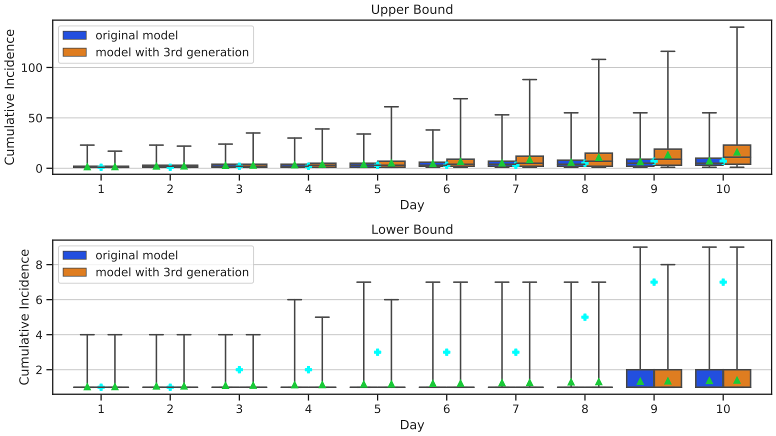

Fig 1. Temporal Comparison of the Original Model and a Model with Third Generation Cases in the Tianjin Office Outbreak. Boxes represent the interquartile range, while whiskers represent the range of estimates from the model simulations. Green triangles indicate the averages of the distribution. Cyan diamonds represent the observations. Upper and lower bound models represent the different set of parameters as described in the main text.

The observations lie within the range of estimates from both models in Fig 1.

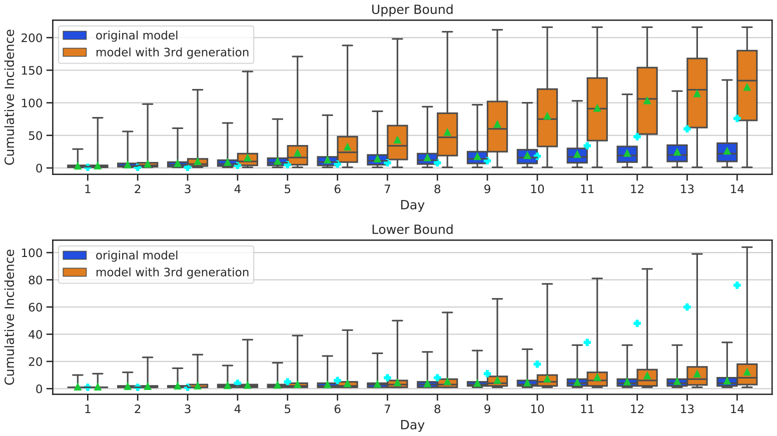

Fig 2. Temporal Comparison of the Original Model and a Model with Third Generation Cases in the Korean Call Center Outbreak. Boxes represent the interquartile range, while whiskers represent the range of estimates from the model simulations. Green triangles indicate the averages of the distribution. Cyan diamonds represent the observations. Upper and lower bound models represent the different set of parameters as described in the main text.

Korean Call Center Outbreak

As shown in Fig 2, the observations lie within the range of estimates from upper bound model. In the lower bound model, the observations exceed the range of the original model starting on the 11th day.

Conclusions

In this analysis, we compared the observational incidences with the original model estimates and those from a preliminary model with third generation cases. The preliminary model has not been validated. Both models have similar interquartile cumulative incidence, and within the same order of magnitude as the observations. The differences between the models appear to be higher in the Korean call center outbreak, which is expected given the higher number of employees, higher SAR, and longer forecast period.

We also expect that the underestimation of cumulative incidence by ignoring third-generation cases is somewhat balanced by the overestimation due to first-generation cases being infectious for the entire forecast period. Multiple studies show a highly variable infectious period distribution ranging from 5 to 20 days depending on the viable viral load [2–5]. As a result, we believe that the original model without consideration of third-generation cases may be suitable for short-term forecasts for small populations within workplaces.

References

1. McAloon C, Collins Á, Hunt K, Barber A, Byrne AW, Butler F, et al. Incubation period of COVID-19: a rapid systematic review and meta-analysis of observational research. BMJ Open. 2020;10(8):e039652. doi:10.1136/bmjopen-2020-039652.

2. Wölfel R, Corman VM, Guggemos W, Seilmaier M, Zange S, Müller MA, et al. Virological assessment of hospitalized patients with COVID-2019. Nature. 2020;581(7809):465–469. doi:10.1038/s41586-020-2196-x.

3. Ferretti L, Ledda A, Wymant C, Zhao L, Ledda V, Abeler L, et al. The timing of COVID-19 transmission. medRxiv. 2020; p. 20.

4. Pan Y, Zhang D, Yang P, Poon LLM, Wang Q. Viral load of SARS-CoV-2 in clinical samples. The Lancet Infectious Diseases. 2020;20(4):411–412. doi:10.1016/S1473-3099(20)30113-4.

5. He X, Lau EHY, Wu P, Deng X, Wang J, Hao X, et al. Temporal dynamics in viral shedding and transmissibility of COVID-19. Nature Medicine. 2020;26(5):672–675. doi:10.1038/s41591-020-0869-5.
